# Altered interhemispheric functional connectivity in end-stage renal disease patients receiving hemodialysis without cognitive impairment

**DOI:** 10.3389/fneur.2025.1585354

**Published:** 2025-05-30

**Authors:** Yan Xue, Bo Li, Shaowen Qian, Gang Sun, Fengyu Jia, Kai Liu

**Affiliations:** ^1^Department of Radiology, Affiliated Hospital of Shandong University of Traditional Chinese Medicine, Jinan, China; ^2^Department of Radiology, The 960th Hospital of the People’s Liberation Army Joint Logistics Support Force, Jinan, China; ^3^Department of Nephrology, The 960th Hospital of the People’s Liberation Army Joint Logistics Support Force, Jinan, China

**Keywords:** end-stage renal disease, hemodialysis, voxel-mirrored homotopic connectivity, magnetic resonance imaging, cognition

## Abstract

**Background:**

Previous studies have shown that alterations in brain structure, metabolism, and function are prevalent in patients with end-stage renal disease (ESRD); however, the synchronization of early functional changes between the two functional hemispheres in these patients remains unclear. This study aimed to analyze interhemispheric functional connectivity in patients with ESRD using resting-state fMRI with voxel mirrored homotopic connectivity (VMHC) algorithm.

**Methods:**

The study cohort comprised 36 patients with ESRD receiving hemodialysis without cognitive impairment and 34 matched healthy control (HCs). All participants completed neuropsychological assessments (e.g., Mini-Mental State Examination, Montreal Cognitive Assessment, Self-Rated Anxiety Scale, and Self-Rated Depression Scale) prior to MR scanning, and all patients underwent laboratory tests.

**Results:**

Compared with HCs, patients with ESRD exhibited significantly decreased VMHC values in bilateral regions including the inferior parietal lobule/angular gyrus, superior temporal gyrus, insula, precentral gyrus, middle occipital gyrus, calcarine/cuneus, and lingual gyrus. No brain regions showed increased VMHC values. Although patients with ESRD had no clinically significant cognitive impairment, their performance on neuropsychological tests was significantly worse than that of HCs (all *p* =0.001). Notably, no correlations were observed between VMHC values and neuropsychological test scores or clinical indicators in patients with ESRD (all *p* > 0.05).

**Conclusion:**

Our findings suggest that interhemispheric connectivity is impaired in patients with ESRD without cognitive impairment, providing novel insights into early-stage neural abnormalities in this population.

## 1 Introduction

End-stage renal disease (ESRD), defined as chronic kidney disease (CKD) stage 5, occurs when the kidneys permanently function at less than 10% of their capacity (1). Patients undergoing chronic hemodialysis frequently exhibit neurologic and psychiatric symptoms such as memory disturbance, impaired attention, slow motor performance, severe depression, and anxiety disorders, which reduce their quality of life and increase health care costs (2–5). Early identification of these cerebral abnormalities is critical for improving clinical management and reducing mortality.

Neuroimaging technology provides an important tool to reveal the mechanism of brain abnormalities in patients with ESRD. Diffusion tensor imaging (DTI) has demonstrated widespread and symmetrical disruption of white matter integrity in patients with ESRD (6–8); Voxel-based morphometry (VBM) has revealed significant gray matter volume loss in bilateral occipital lobes, superior temporal gyrus, calcarine, lingual, and cuneus (9, 10); Arterial spin labeling (ASL) has identified abnormal cerebral blood flow predominantly in bilateral limbic systems and temporal lobes (11). Resting-state functional magnetic resonance imaging (rs-fMRI) using regional homogeneity (ReHo) and amplitude of low-frequency fluctuations (ALFF) has further shown decreased intrinsic neural activity in bilateral cerebral hemispheres of patients with ESRD (12–15). These findings collectively suggest that structural, metabolic, and functional brain abnormalities are common in ESRD, with bilateral hemispheric involvement may be a typical feature. However, existing studies have not systematically evaluated the breakdown mode of interhemispheric functional connectivity in patient with ESRD, which is crucial to understanding the cross-hemispheric integration mechanism of neurocognitive decline. Interhemispheric connectivity, which reflects information exchange and integration between bilateral hemispheres, serves as the neural basis for cognitive, behavioral, and emotional functions (16).

Weakening of functional connectivity is directly associated with neuropsychological impairment in patients with ESRD (17–19). Previous rs-fMRI studies have identified abnormal functional connectivity in networks such as the default-mode network (DMN), salience network (SN), sensorimotor network (SMN) in patient with ESRD (9, 20). However, the alterations may have an extensive and variable pattern based on different analytical algorithms such as global functional connectivity analysis (21), independent component analysis (18), graph theory-based network analysis (20, 22–24), and seed-based network analysis (9, 20). Moreover, existing studies has predominantly focused on unilateral or local networks, and the exploration of bilateral hemispheric functional coordination is still insufficient. The primary challenge lies in directly characterizing ESRD-related interhemispheric dysfunction and its association with neuropsychological deficits, given the limitations of prior methodologies. Voxel-mirrored homotopic connectivity (VMHC), a new rs-fMRI method developed by Zuo et al. (25), provides a direct method for quantifying hemispheric interaction by identifying the temporal correlation of the low-frequency blood oxygen level-dependent (BOLD) signals between the two mirrored voxels across the contralateral hemispheres. This technique has been extensively applied to investigate interhemispheric functional coordination in neurologic and psychiatric disorders, including Major depressive disorder (26, 27), Generalized anxiety disorder (28), Parkinson disease (29, 30), and Alzheimer’s disease (31). However, its utility remains unexplored in ESRD. The purpose of this study was to: (a) identify the spatial distribution characteristics of VMHC abnormalities in patients with ESRD; (b) explore the association between VMHC changes and neurocognitive scores and clinical indicators; (c) verify the potential of VMHC as a neuroimaging biomarker for early brain dysfunction in ESRD.

## 2 Materials and methods

### 2.1 Subjects

This cross-sectional study was approved by the Ethics Committee of the 960th Hospital of the People’s Liberation Army (PLA) and written informed consent was obtained from each subject before the study. A total of 48 right-handed patients with ESRD without cognitive impairment were recruited from the hemodialysis center of the 960th Hospital of the PLA and participated in MR scanning on a non-dialysis day. The exclusion criteria were applied as follows: (a) age ≤18 years, (b) dialysis duration <6 months, (c) history of drug or alcohol abuse, (d) history of psychiatric or neurologic disorders, (e) moderate or severe depressive and anxiety mood (20), (f) diabetes history (24, 32), (g) visible brain lesions (e.g., tumor, stroke, infarction) on conventional MR imaging, and (h) head motion >1 mm or 1° during scanning. Twelve patients with ESRD were excluded because of visible infarction (*n* = 2), significant depressive mood (*n* = 2), diabetes (*n* = 2) and excessive head motion (*n* = 6). Finally, the remaining 36 patients with ESRD (23 males, 13 females; age range: 27–66 years; mean ± SD: 47.83 ± 12.64 years) were included in the final analysis.

A total of 40 right-handed, age- and sex-matched healthy subjects (HCs) were recruited from the local community. The exclusion criteria were similar to those used for patients with ESRD. Accordingly, six subjects were excluded due to excessive head motion. The remaining 34 healthy subjects (22 males, 12 females; age range 23–72 years; mean age 46.94 ± 16.86 years) were included in the final analysis.

Prior to MR scanning, all subjects completed a comprehensive assessment protocol including demographic data collection (sex, age, and education level), dialysis duration, and both cognitive and affective evaluations. Cognitive function was assessed using two well-validated instruments, namely the Chinese version of the Mini-Mental State Examination (MMSE) test and the Montreal Cognitive Assessment (MoCA) test (33, 34). We strictly followed the questionnaire instructions to define the cognitive levels of the subjects. Anxiety and depression were measured using the Self-Rated Anxiety Scale (SAS) (35) and Self-Rated Depression Scale (SDS) (36), respectively. The Chinese versions have been widely used and demonstrates adequate reliability and validity in previous studies (37, 38). A routine laboratory test for the patients with ESRD was done using DXC800 Automatic biochemical analyzer (Beckman Coulter, California, United States) within a day.

### 2.2 Data acquisition

The MR data were collected using a 3.0 T MR system (MR750, General Electric, Milwaukee, Wisconsin, United States) with an eight-channel head coil. Each scan consisted of 200 EPI functional volumes with the following parameters: TR 2000 ms, TE 35 ms, slice thickness 4 mm, slices 35, flip angle (FA) 90°, matrix 64 × 64, and a field of view (FOV) 24 × 24 cm^2^. A high-resolution T1-weighted sequence was obtained: TR, 8.2 ms; TE 3.2 ms, slices 132, slice thickness 1.0 mm, FOV 24 × 24 cm^2^, and FA 12°. Additionally, the conventional imaging was performed by an axial T1- and T2 weighted spin-echo sequence and T2-fluid attenuated inversion recovery sequence to exclude subjects with visible brain lesions.

### 2.3 Data processing

Resting-state imaging data were preprocessed using the Data Processing Assistant for Resting-state fMRI (DPARSF) and Resting-state fMRI Data Analysis Toolkit (REST),^1^ which were carried out based on Statistical Parametric Mapping (SPM8; http://www.fil.ion.ucl.ac.uk/spm). The first 10 time points were discarded to guarantee the stability of the fMRI signals. The remaining images were corrected for slice timing and head motion. Then, T1 images were co-registered to the realigned functional images and segmented to gray matter, white matter, and cerebrospinal fluid. The resulting images were registered to standard Montreal Neurological Institute (MNI) space. The functional images were also normalized to the MNI space and resampled to 3 × 3 × 3 mm^3^. The generated images were spatially smoothed with a four mm full-width-half-maximum (FWHM), temporally band-pass filtered (0.01–0.08 Hz), and linearly detrended removal to reduce low-frequency drift and high-frequency noise. Finally, several sources of spurious covariates along with their temporal derivatives were then removed from the data by using linear regression, including six head motion parameters obtained by rigid body correction, the signal from a ventricular region of interest, and the signal from a region centered in the white matter.

### 2.4 Interhemispheric correlation

The VMHC computation was performed with REST software. For each subject, the homotopic resting-state functional connectivity was calculated as the Pearson correlation coefficient between each voxel’s residual time series and that of its mirrored interhemispheric counterpart. The correlation coefficients were then Fisher z-transformed to improve normality. The resultant values generated VMHC maps and were used for subsequent group-level analyses. The details of VMHC obtainment have been expounded in a previous study (25).

### 2.5 Statistical analysis

The demographic and clinical data were analyzed by SPSS version 22 (SPSS Inc., Chicago, Illinois, United States). For continuous variables (e.g., age, education, MMSE, MoCA, SDS, SAS scores), two-sample independent Student’s t-tests were applied to compare group differences between ESRD group and HC group, as these variables met normality assumptions (assessed by Shapiro–Wilk tests) and homogeneity of variance (confirmed by Levene’s test). Categorical variables (e.g., sex) were analyzed using the Chi-squared test, which is appropriate for comparing proportions between independent groups. Results were reported as mean ± SD for continuous data and frequencies for categorical data, with statistical significance defined as *p <* 0.05.

Resting-state fMRI data was analyzed using REST software. Group-level differences in VMHC maps were examined through voxel-wise two-tailed t-test, with age and sex included as covariates to control for potential confounding effects. Multiple comparisons were addressed through false discovery rate (FDR) correction (Q value < 0.05, cluster size > 30 voxels). Brain areas exhibiting significant VMHC differences between groups were identified as regions of interest (ROIs), and mean VMHC values were separately extracted from ROIs for further analysis. Pearson correlation analysis was calculated between VMHC values and clinical variables in patients with ESRD, as both variables were continuous and normally distributed. All *p <* 0.05 were regarded as statistically significant.

## 3 Results

### 3.1 Demographics and clinical characteristics

The demographic and clinical data of all subjects are summarized in Table 1. There were no significant differences in age (*p* = 0.804), sex (*p* = 0.943), and education level (*p* = 0.949) between the ESRD group and the HC group. While patients with ESRD exhibited no clinically overt cognitive impairment, both MMSE and MoCA scores of the ESRD group were significantly lower than those of the HC group (*p* = 0.001). Notably, both SAS and SDS scores were significantly higher in the ESRD groups compared to the HC group (all *p* = 0.001).

**Table 1:** Demographic and clinical characteristics of patients with ESRD and HCs.

Variables	ESRD (*n* = 36)	HC (*n* = 34)	t/x^2^	*p*-value
Demographic data				
Age (years)	47.83 ± 12.64	46.94 ± 16.86	0.249	0.804^a^
Sex (male/female)	23/13	22/12	0.005	0.943^b^
Education (years)	11.69 ± 2.51	11.73 ± 2.82	−0.064	0.949^a^
Hypertension	17/36			
Dialysis duration (months)	71.50 ± 66.05			
Neuropsychological test				
MMSE (score)	28.25 ± 1.11	29.14 ± 1.10	−3.395	0.001^a^
MoCA (score)	26.25 ± 0.87	28.03 ± 1.40	−6.325	0.001^a^
SDS (score)	43.72 ± 8.06	32.50 ± 6.37	6.430	0.001^a^
SAS (score)	41.83 ± 7.53	31.82 ± 7.27	5.652	0.001^a^
Laboratory data				
Hemoglobin (g/L)	103.22 ± 15.00			
Albumin (g/L)	39.41 ± 4.04			
Calcium (mmol/L)	2.32 ± 0.25			
Phosphorus (mmol/L)	1.76 ± 0.50			

a The *p* values were obtained by student’s t test.

b The *p* value was obtained by Chi-square test.

ESRD, end-stage renal disease; HC, healthy control; MMSE, Mini-Mental State Examination; MoCA, Montreal Cognitive Assessment; SDS, Self-Rated Depression Scale; SAS, Self-Rated Anxiety Scale.

### 3.2 VMHC differences

**Figure 1:**
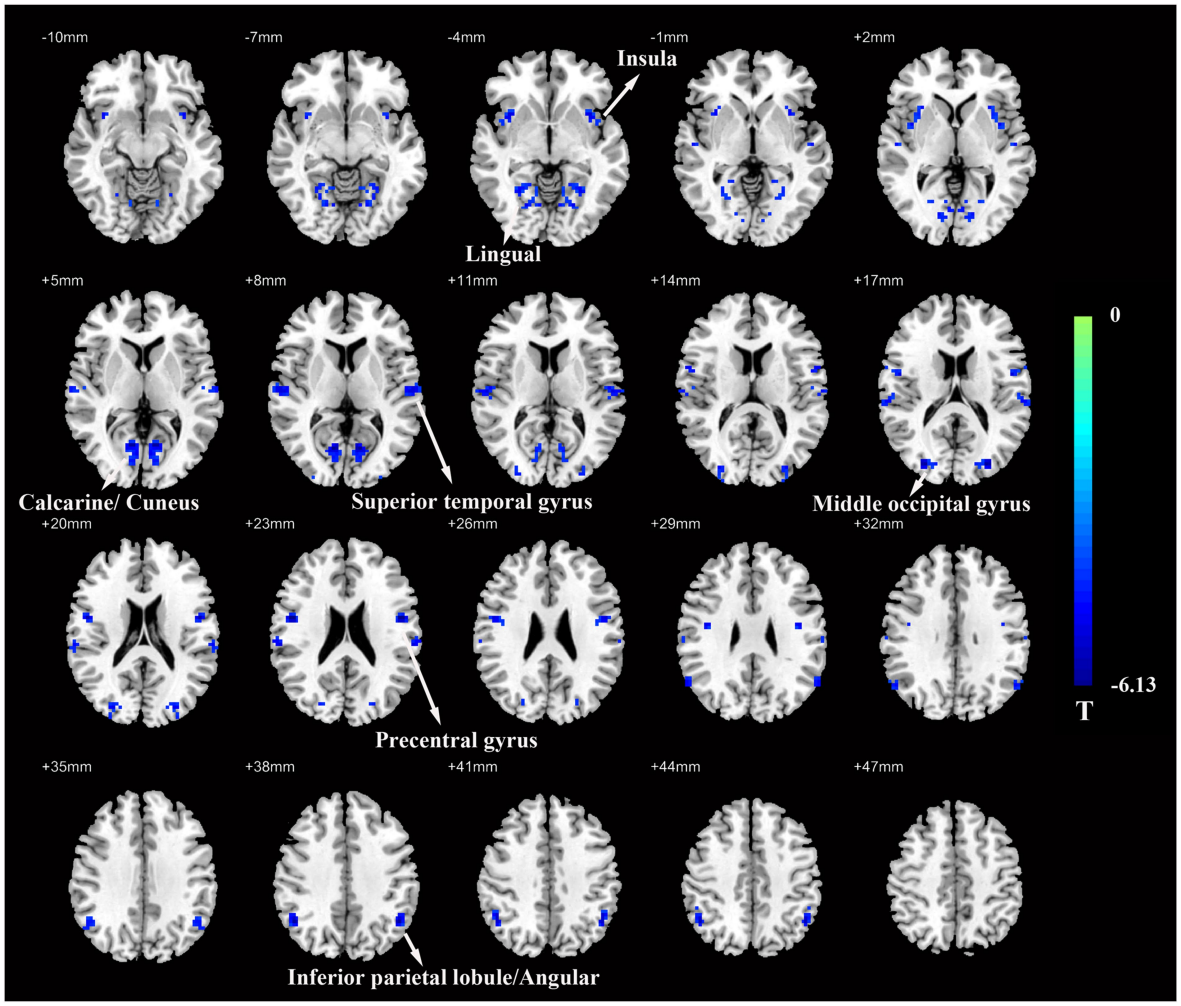
Voxel-mirrored homotopic connectivity (VMHC) map of patients with ESRD and HCs. Compare with HCs, patients with ESRD showed significantly decreased VMHC values in the inferior parietal lobule/angular, superior temporal gyrus, insula, precentral gyrus, middle occipital gyrus, calcarine/cuneus, and lingual. Color bars indicate the t values from the global voxel-based two-sample t-test (Q value < 0.05, FDR corrected). ESRD, end-stage renal disease; HC, healthy control.

Group comparison results of VMHC are presented in Figure 1 and Table 2. As compared with HCs, patients with ESRD showed significantly decreased VMHC values mainly located in the bilateral inferior parietal lobule (IPL)/ angular, bilateral superior temporal gyrus (STG), bilateral insula, bilateral precentral gyrus (PreCG), bilateral middle occipital gyrus (MOG), bilateral calcarine/cuneus, and bilateral lingual. Compared with HCs, there was no increased VMHC value in any region in patients with ESRD.

**Table 2:** Brain regions with significant VMHC differences between patients with ESRD and HCs.

Regions	BA	MNI coordinates			Voxels	Tmax
		x	y	z		
Precentral gyrus	6/48	±51	−3	24	41	−6.134
Superior temporal gyrus	22/48	±57	−18	9	63	−5.157
Inferior parietal lobule/Angular	39/40	±48	−57	39	52	−5.817
Middle occipital gyrus	19	±27	−84	18	36	−5.836
Insula	48	±36	12	−3	33	−5.003
Calcarine/Cuneus	17	±12	−69	9	62	−5.956
Lingual	18/19	±21	−69	−3	51	−4.901

BA, brodmann area; MNI, montreal neurological institute; VMHC, voxel-mirrored homotopic connectivity; ESRD, end-stage renal disease; HC, healthy control.

### 3.3 Correlation analysis

There were no significant correlations between VMHC values and MMSE score, MoCA score, SAS score, SDS score, dialysis duration, hemoglobin, albumin, calcium, phosphorus level in patients with ESRD (all *p >* 0.05).

## 4 Discussion

Our study provides novel evidence of disrupted interhemispheric functional coordination in patients with ESRD receiving hemodialysis without cognitive impairment. Through VMHC analysis, we identified significant reductions in interhemispheric connectivity across multiple brain regions, predominantly involving the IPL, STG, insula, PreCG, MOG, and calcarine/lingual/cuneus regions. Some of these affected regions constitute core nodes of three major neurocognitive networks: the DMN the SN and the SMN. Critically, our findings reveal that interhemispheric dysconnectivity in these regions emerges prior to detectable cognitive deficits on standard clinical assessments. This observation positions VMHC as a sensitive neuroimaging biomarker capable of detecting preclinical network-level disruptions in ESRD.

Our study revealed significantly decreased VMHC values in DMN related regions including IPL and STG in patients with ESRD compared with HCs. The DMN, which exhibits increased activity in the resting state and suspends during cognitive tasks, plays crucial roles in different high-level cognitive functions such as memory, visual and auditory attention, motor activity, and language processing (18). Numerous studies based on various rs-fMRI analytical algorithms have demonstrated reduced neural activity in DMN-related regions among patients with ESRD. For example, the ReHo algorithm revealed decreased ReHo values in multiple areas of bilateral frontal, parietal, and temporal lobes in patients with ESRD, with a more pronounced reduction of ReHo in the DMN among hemodialysis patients compared to non-dialysis patients (12), while the ReHo values of the DMN in patients with mild cognitive impairment were significantly lower than those without cognitive impairment (14). The ALFF algorithm also observed that the ALFF values in the DMN region of patients with ESRD were decreased, and the ALFF value of IPL in peritoneal dialysis (PD) patients was lower than those of non-dialysis patients (15). These results suggested that patients with ESRD, especially those receiving dialysis treatment, are prone to abnormal brain activity in the DMN, and the degree of DMN damage may be an important risk factor for aggravating cognitive dysfunction. The clinical relevance of DMN abnormalities is further supported by correlations between neuroimaging metrics and cognitive performance. Previous studies reported positive associations between digit-symbol test (DST) scores—a test of attention and visual memory domains—and both ALFF/ReHo values in the IPL in patients with ESRD (14, 15). Although our cohort maintained preserved daily function without clinical cognitive impairment, the observed VMHC reductions in IPL and STG suggest that interhemispheric dysconnectivity within DMN regions may precede overt cognitive decline. This interpretation is reinforced by prior rs-fMRI investigations demonstrating altered DMN connectivity patterns in neurologically asymptomatic patients with ESRD (17, 20). Our VMHC findings provide further evidence from an interhemispheric functional network integrity view that the interhemispheric connectivity within the DMN is also impaired in ESRD patients without cognitive impairment.

The current study also found decreased VMHC values in the insula of patients with ESRD. This finding aligns with previous study demonstrating reduced functional connectivity in the bilateral insula of hemodialysis patients with ESRD using global functional connectivity analysis (21). The insula receives and integrates the interoceptive signals, and it is also engaged in the processing of negative emotions and anticipating pain (39). Affective disorders are common clinical manifestations in patients with ESRD. For example, the prevalence of depression in this population is as high as 20–25% (22). Although only two of the participants in our cohort reported significant depression mood, patients with ESRD showed an increase in SDS and SAS scores as compared with HCs, which was consistent with a previous study (15). Generally, decreased VMHC in the insula has been found in patients with major depressive disorder and generalized anxiety disorder (28, 40). This indicates that the impairment of interhemispheric communication in this region leads to emotional dysregulation. Combined with previous findings, the impaired interhemispheric synchronous of the insula in our study may reflect a functional decline in the processing of emotion-related functions, which might partially contribute to the emotional symptoms observed in patients with ESRD. Furthermore, the insula is also a core region of the SN, and the abnormalities in the SN are associated with neurological, psychological, and behavioral disorders in patients with ESRD. Global functional connectivity density analysis revealed that neurologically asymptomatic patients with ESRD receiving hemodialysis exhibited abnormal intrinsic dysconnectivity pattern of SN regions over bilateral insula and dorsal anterior cingulate cortex (41). Seed-based connectivity analysis also revealed that patients with ESRD receiving PD without cognitive decline exhibited some alterations of SN connectivity (seed at right insula), including increased functional connectivity at the thalamus and decreased functional connectivity at STG, anterior cingulate gyrus, and postcentral gyrus (20). These studies indicate that the SN could be a potential network susceptible to the changes of between-network connectivity in patients with ESRD without cognitive impairment. However, further research is needed to clarify whether dialysis modalities will affect the pattern of connectivity alteration.

In this study, decreased VMHC values were also observed in the bilateral PreCG in patients with ESRD. The PreCG, also known as the primary motor cortex, is responsible for motor control, particularly in complex motor behavior (42). A previous study reported decreased ReHo values in the bilateral PreCG in both hemodialysis and non-dialysis patients with ESRD compared to HCs (12). Furthermore, the decreased ReHo values of the bilateral PreCG were negatively correlated with the Number Connection Test-A (NCT-A), a measure of psychomotor speed in patients with ESRD, suggesting that abnormal brain activity in the bilateral PreCG may be associated with impaired psychomotor performance (14). Decreased interhemispheric connectivity represents insufficient communication between hemispheres and a weak synergistic balance between bilateral brain regions. For instance, the strong association between decreased VMHC in PreCG and motor control deficit in chronic stroke patients and Parkinson’s patients highlights the disruptive effects of inadequate interhemispheric communication on motor control function (43, 44). In patients with ESRD, decreased interhemispheric connectivity in the PreCG may contribute to motor control dysregulation. Interesting, the PreCG is also a key component of the SMN, which is involved in sensory input or motor coordination. A recent study demonstrated alteration of SMN connectivity (seed at right PreCG) including decreased functional connectivity at the insula and parahippocampal gyrus in patients with ESRD undergoing PD without cognitive decline (20). Despite different renal replacement therapy, both studies revealed decreased SMN connectivity. We speculate that SMN connectivity may represent a potential brain functional network susceptible to the effects of ESRD.

Another important finding was the observation of decreased VMHC values in multiple visual regions of the occipital lobe including MOG, cuneus, lingual, and calcarine in patients with ESRD. The MOG plays a critical role in face processing perception, essential for social interaction (45), while the calcarine, lingual gyrus, and cuneus are key regions implicated in visual processing and visual pathways (46). Prior studies have demonstrated functional and structural abnormalities in these visual regions in patients with ESRD (10, 14). Neuroimaging evidence further revealed that the ALFF values of bilateral calcarine were negatively correlated with serial dotting test (SDT) scores in patients with ESRD, and the ALFF values of bilateral cuneus were negatively correlated with line-tracing test (LTT) scores, indicating that visual cortical dysfunction may contribute to visual-motor deficits (15). The visual information from each hemi field is transmitted to the contralateral side of the primary visual cortex simultaneously, and this information needs to be integrated into the bilateral cortical hemispheres (47). The activation of interhemispheric connections in the visual cortex is very important for the early stage of visual information processing (48). Most of these occipital lobes and visual signal encoding functional areas are related to visual information processing and modulation of top-down visuospatial selective attention. The decreased VMHC in the visual cortex could disorder the visual information exchange and processing between the bilateral hemispheres, which may manifest as a visual cognitive dysfunction.

In addition, we performed a correlation analysis between the VMHC values and clinical variables, however, no significant correlation was observed in patients with ESRD. The potential reasons for this are as follows: first, the relatively small sample size may have resulted in insufficient statistical power. Second, neuropsychological assessments, which are self-rating scales, may be affected by some confounders such as educational levels, intelligence, illness duration, and social environment. In this study, certain indicators, including the MMSE and MoCA, fell within the normal range, which likely contributed to the absence of a significant correlation. Third, given that ESRD is a chronic and progressive condition, patients often receive multiple treatments for comorbidities (e.g., anemia, hypertension), which may induce fluctuations in biochemical markers.

While our study provides novel insights into interhemispheric dysconnectivity patterns in ESRD patients without cognitive impairment, several methodological considerations should be acknowledged. The cross-sectional design precludes causal inferences regarding the temporal progression of VMHC alterations. Additionally, the modest sample size may have limited both our ability to detect subtle clinical correlations and the statistical power to demonstrate differences between groups. Nevertheless, our rigorous matching of demographic variables between groups and the utilization of novel analytical approaches strengthen the validity of the observed interhemispheric connectivity patterns. Future longitudinal studies with larger cohorts should investigate whether the reductions of VMHC in specific brain regions in early stages can predict subsequent cognitive decline among patients with ESRD. Furthermore, comparative analyses across dialysis modalities (e.g., hemodialysis vs. peritoneal dialysis) could elucidate treatment-specific effects on functional connectivity. Finally, by integrating VMHC with multi-modal neuroimaging data (e.g., diffusion tensor imaging for structural connectivity) and supplementing a battery of neuropsychological tests (e.g., NCT-A, DST, SDT, LTT, and trial marking test-A&B), this combined methodology may provide a more comprehensive understanding of brain network-level disruption and related cognitive impairment mechanisms in patients with ESRD.

## 5 Conclusion

This study demonstrates that patients with ESRD receiving hemodialysis without clinical cognitive impairment exhibit widespread interhemispheric connectivity deficits in key functional networks including the DMN, SN, and SMN. The observed VMHC reductions in regions governing cognitive integration (IPL/STG), emotional processing (insula), motor coordination (PreCG), and visual information transfer (occipital lobe) suggest that interhemispheric dysconnectivity may represent an early neurological feature of subclinical brain dysfunction in ESRD. Crucially, our findings confirm that the primary research objective—to characterize interhemispheric functional alterations preceding overt cognitive decline in ESRD—has been achieved. These results advance our understanding of ESRD-related neuropathology by identifying VMHC as a sensitive neuroimaging biomarker for detecting preclinical network disruptions.

## Data Availability

The original contributions presented in the study are included in the article/supplementary material, further inquiries can be directed to the corresponding author.

## Footnotes

^1^ http://www.restfmri.net
